# Transcriptomic analysis of salt tolerance-associated genes and diversity analysis using indel markers in yardlong bean (*Vigna unguiculata* ssp. *sesquipedialis*)

**DOI:** 10.1186/s12863-021-00989-w

**Published:** 2021-09-16

**Authors:** Hongmei Zhang, Wenjing Xu, Huatao Chen, Jingbin Chen, Xiaoqing Liu, Xin Chen, Shouping Yang

**Affiliations:** 1 Soybean Research Institute of Nanjing Agricultural University/National Center for Soybean Improvement/National Key Laboratory for Crop Genetics and Germplasm Enhancement, Nanjing, 210095 Jiangsu China; 2 Institute of Industrial Crops, Jiangsu Academy of Agricultural Sciences/Jiangsu Key Laboratory for Horticultural Crop Genetic Improvement, No. 50, Zhongling Street, Nanjing, 210014 Jiangsu China; 3 College of Horticulture, Nanjing Agricultural University, Nanjing, 210095 Jiangsu China

**Keywords:** DEGs, Indels, LR48 transcription factor, Salt stress, Transcriptome, Yardlong bean

## Abstract

**Background:**

High salinity is a devastating abiotic stresses for crops. To understand the molecular basis of salinity stress in yardlong bean (*Vigna unguiculata ssp. sesquipedalis*), and to develop robust markers for improving this trait in germplasm, whole transcriptome RNA sequencing (RNA-seq) was conducted to compare the salt-tolerant variety Suzi 41 and salt-sensitive variety Sujiang 1419 under normal and salt stress conditions.

**Results:**

Compared with controls, 417 differentially expressed genes (DEGs) were identified under exposure to high salinity, including 42 up- and 11 down-regulated DEGs in salt-tolerant Suzi 41 and 186 up- and 197 down-regulated genes in salt-sensitive Sujiang 1419, validated by qRT-PCR. DEGs were enriched in “Glycolysis/Gluconeogenesis” (ko00010), “Cutin, suberine and wax biosynthesis” (ko00073), and “phenylpropanoid biosynthesis” (ko00940) in Sujiang 1419, although “cysteine/methionine metabolism” (ko00270) was the only pathway significantly enriched in salt-tolerant Suzi 41. Notably, *AP2/ERF*, *LR48*, *WRKY*, and *bHLH* family transcription factors (TFs) were up-regulated under high salt conditions. Genetic diversity analysis of 84 yardlong bean accessions using 26 InDel markers developed here could distinguish salt-tolerant and salt-sensitive varieties.

**Conclusions:**

These findings show a limited set of DEGs, primarily TFs, respond to salinity stress in *V. unguiculata*, and that these InDels associated with salt-inducible loci are reliable for diversity analysis.

**Supplementary Information:**

The online version contains supplementary material available at 10.1186/s12863-021-00989-w.

## Background

The legume cowpea (*Vigna unguiculata* L. Walp.) is the fifth most widely consumed plant-based source of protein and soluble fiber [1], and the *sesquipedalis* subspecies, i.e.*,* asparagus bean or ‘yardlong’ bean, is cultivated as a prized vegetable among eastern and southern Asian countries [2, 3]. Abiotic stress induced by high salinity can lead to major reductions in growth, yield, and quality, so improvement to salt tolerance represents an urgent priority for yardlong bean breeding programs. Uncovering the molecular mechanisms underlying plant response to salt stress can enable development of salt-tolerant yardlong bean cultivars. To date, several mechanisms have been identified across a range of model plants and crops for their role in tolerance to high salinity, including modulation of ion and osmotic homeostasis, stress-induced cellular repair pathways, and alternative growth regulatory pathways that circumvent stress response signaling [4].

Salt-tolerant plants characteristically exhibit adaptive maintenance of intracellular ion homeostasis, and in particular, the salt overly sensitive (SOS) pathway has been implicated in maintaining a K^+^/ Na^+^ ratio essential for growth under high salinity conditions. The SOS pathway involves regulation of ion transport by the SOS1 Na^+^/H^+^ plasma membrane antiporter, which is activated via the SOS3 calcium sensor and SOS2 Ser/Thr protein kinase [5, 6]. Other known regulators of ion transport and exclusion include *Arabidopsis* K^+^ transporter1 (AKT1), Na^+^/H^+^ exchangers (NHXs), high sodium affinity transporter (HKT), and other plasma membrane proteins (PMP), all of which may be activated under exposure to high salinity to ensure an ion balance that allows continued cellular function [4, 7]. In addition to transporters, transcription factors from several families participate in ion homeostasis and salt tolerance through regulation of signal transduction pathways and downstream transporters, such as apetala2/ethylene responsive factor (AP2/ERF), dehydration responsive element binding protein (DREB), basic leucine zipper domain (bZIP), WRKY, and MYB, among others [8–12].

Osmotic homeostasis is also reportedly regulated also by different MAP kinase (MAPK) signal cascade-mediated programmed responses that control osmotic homeostasis, for example through vacuolar Na^+^ sequestration or via synthesis and accumulation of biocompatible osmolytes [13–15]. In addition, salt stress is typically accompanied by reactive oxygen species (ROS) burst that can disrupt metabolic activity or damage lipid membranes and DNA [16]. Plants have thus evolved multiple enzymes to scavenge and detoxify ROSs, minimize their damage, and enhance repair of cellular damage including superoxide dismutase, ascorbate peroxidase, catalase, guaiacolperoxidase, and others. Furthermore, plants synthesize metabolites and small molecules that also function as antioxidants, such as ascorbic acid, alkaloids, carotenoids, flavonoids, phenolic compounds, and tocopherol, etc. [17, 18].

Since its introduction from Africa, yardlong bean has been increasingly selected for stress-resistant phenotypes suitable for cultivation in Asia. Chen et al. (2007) and Murilloamador et al. (2006) both identified salt tolerant *sequipedalis* genotypes [19, 20], while more recently, Xu and colleagues used genome-wide association study (GWAS) to reveal thirty-nine SNP loci associated with drought resistance [21]. Tan et al. (2016) identified several genes that were differentially expressed genes (DEGs) between cold-tolerant and -sensitive yardlong bean cultivars, while Pan et al. (2019) found 216 and 127 salt stress-associated DEGs in roots and leaves, respectively, six of which were linked to 17 salt tolerance-associated SNP markers [22, 23]. More recently, other QTLs associated with salt tolerance in yardlong bean were mapped using a population generated by crossing Suzi 41 (salt tolerant) × Sujiang 1419 (salt sensitive) [24]. Completion of the yardlong bean genome and the relatively low cost of re-sequencing have enabled further development of SNP/InDel markers in *sesquipedalis* for use in breeding and genetic analysis, as has been widely reported in common bean and mungbean [25–27].

In this study, RNA-seq analysis was used to compare transcriptional responses of two varieties of yardlong bean, Suzi 41 (salt-tolerant) and Sujiang 1419 (salt-sensitive), to identify the regulatory and metabolic pathways mediating salt stress response in this high value crop. A set of DEGs encoding transcription factors were identified which could regulate downstream pathways necessary for salt tolerance. In addition, KEGG and GO analysis were performed to predict the putative functions of DEGs, and then the differences in transcriptional regulation between the salt-tolerant and -sensitive varieties were compared. Importantly, this study developed a set of informative and reliable salt stress-specific InDel markers based on high throughput sequencing and revealed considerable genetic diversity in *V. unguiculata*. This work provides insight into the basic mechanisms underlying salt tolerance, as well as tools for applied research necessary for improvement of yardlong bean varieties for cultivation in high saline soils.

## Results

### Transcriptome sequencing and discovery of novel transcripts

**Fig. 1:**
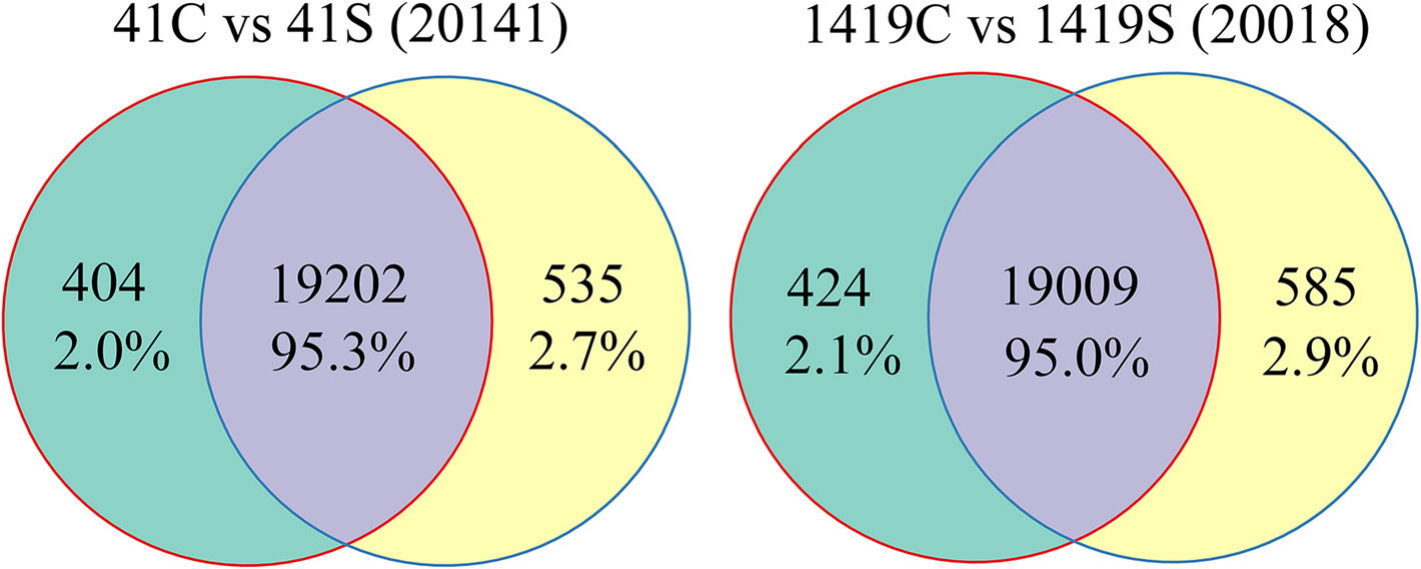
Venn diagrams showing the number of mapped genes shared by each combination of library pairs. 41C, Suzi 41 control; 41S, Suzi 41 salt-stressed; 1419C, Sujiang 1419 control; 1419S, Sujiang 1419 salt-stressed

The Illumina HiSeq™ 2000 platform was used to sequence Suzi 41 and Sujiang 1419 transcriptomes in yardlong bean that were treated under high salt stress conditions (41S and 1419S) to compare with those of unstressed plants (41C and 1419C) in order to identify differences in their transcriptional responses to high salinity by these two phenotypically different varieties. After removing low-quality sequences and trimming adapter sequences, ~ 24 million paired-end reads were generated from each of the cDNA libraries with an average GC content of 45.65%. All clean reads were matched to the *Vigna unguiculata* reference genome by TopHat software. As a result, about 43 million mapped reads were obtained for each line of Suzi 41 and Sujiang 1419, with an average matching rate of 89.83% (Supplementary Table S1). Most (99.56–99.72%) of the reads with matches were unique reads (matching only one yardlong bean locus), while the remainder (~ 0.28–0.44%) were non-unique (matching more than one yardlong bean locus) or unaligned. For more detailed investigation of gene expression in the different treatments, only unique reads were used in the analysis. In both control and salt stress treatments, the numbers of mapped genes in Suzi 41 (19,606 and 19,737 genes) were found to be similar to those in Sujiang 1419 (19,433 and 19,594 genes, respectively). The mapped genes among the four treatments (41C, 41S, 1419C, and 1419S) were further compared, and ~ 95% of them were present in at least two treatments (Fig. 1).

Identification of novel transcript isoforms has emerged as one of the major advantages of RNA-seq analysis. This study revealed a total of 563 novel transcript isoforms in Suzi 41 and Sujiang 1419 yardlong bean varieties. Comparison of transcriptomic reads with the *Vigna unguiculata* reference genome revealed that most new genes (562; 99.82%) were annotated by nr, followed by GO (361; 64.12%) and Swissprot (319; 56.66%). Only 64 (11.37%) DEGs were annotated with COG (Supplementary Table S2). Respectively, 299 (53.11%), 243 (43.16%) and 90 (15.99%) DEGs were annotated with Pfam, KOG and KEGG. Although the novel transcript isoforms will be validated in future experiments, they were included in further analyses for preliminary functional characterization and investigation of their putative role in abiotic stress responses.

### Differential gene expression in response to salt-stress treatments

**Fig. 2:**
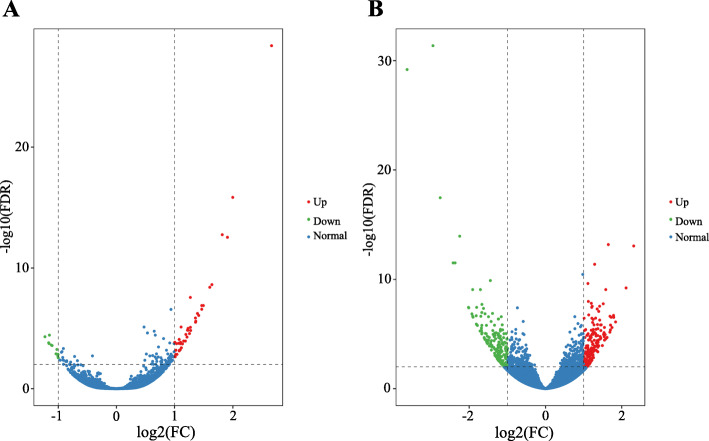
Volcano plots of DEGs under salt stress for **A** Suzi 41 and **B** Sujiang 1419

Differential gene expression analysis of Suzi 41 and Sujiang 1419 genotypes revealed 390 differentially expressed genes (DEGs) in the salt stress vs control comparison (Fig. 2, Supplementary Table S3). There were 42 and 11 genes identified as being up- and down-regulated in the salt-tolerant genotype Suzi 41, respectively, and173 and 183 genes identified in the salt-sensitive genotype Sujiang 1419, respectively. There were more DEGs in Sujiang 1419 than in Suzi 41.

Under salt tolerance, a number of genes were expressed only in the salt-tolerant genotype. In Suzi 41, there were 32 and 2 DEGs were identified as being up- and down-regulated that were not also differentially expressed in Sujiang 1419 (Supplementary Table S4). Interestingly, the most highly up-regulated of these are LR48 transcription factors including (with Log_2_ fold change) *Vigun02g152900* (2.00), *Vigun10g012000* (1.91), *Vigun10g011500* (1.82), and *Vigun10g011900* (1.60), as well as a PR-4-like pathogenesis-related protein *Vigun06g113200* (1.64). The two differentially down-regulated genes include a LR48 protein, *Vigun05g219700* (− 1.17), and a WAT1-related protein *Vigun06g228300* (− 1.02). The prevalence of transcription factors among DEGs strongly suggests that these genes are responsible for promoting salt tolerance in Suzi 41, and may serve as potentially strong candidates for further elucidation of the mechanisms underlying salt tolerance in yardlong bean.

### Functional annotation and classification of DEGs

Ten DEGs were up-regulated and 9 DEGs were down-regulated in both varieties (Supplementary Table S5), suggesting that these genes are differentially expressed specifically under salt stress in both varieties. Among the most highly up-regulated overlapping DEGs are a hypothetical bZIP LR48 gene (annotated in Phytozome as senescence-associated protein PF06911) (*Vigun11g188200*), as well as several other transcription factors, and a predicted peroxidase 21 (*Vigun07g080600*). Among the down-regulated genes found in both varieties, metalloendoproteinase 1-like protein (*Vigun02g070900*) and an alcohol dehydrogenase (*Vigun09g123700*), both with a Log_2_ fold change of ~ − 1.0 in susceptible and tolerant yardlong bean varieties, and several hypothetical LR48 transcription factors were identified. These genes, which were differentially expressed under salt stress in both varieties may serve as a basis for identifying target genes for molecular breeding to improve salt tolerance in yardlong bean.

**Fig. 3:**
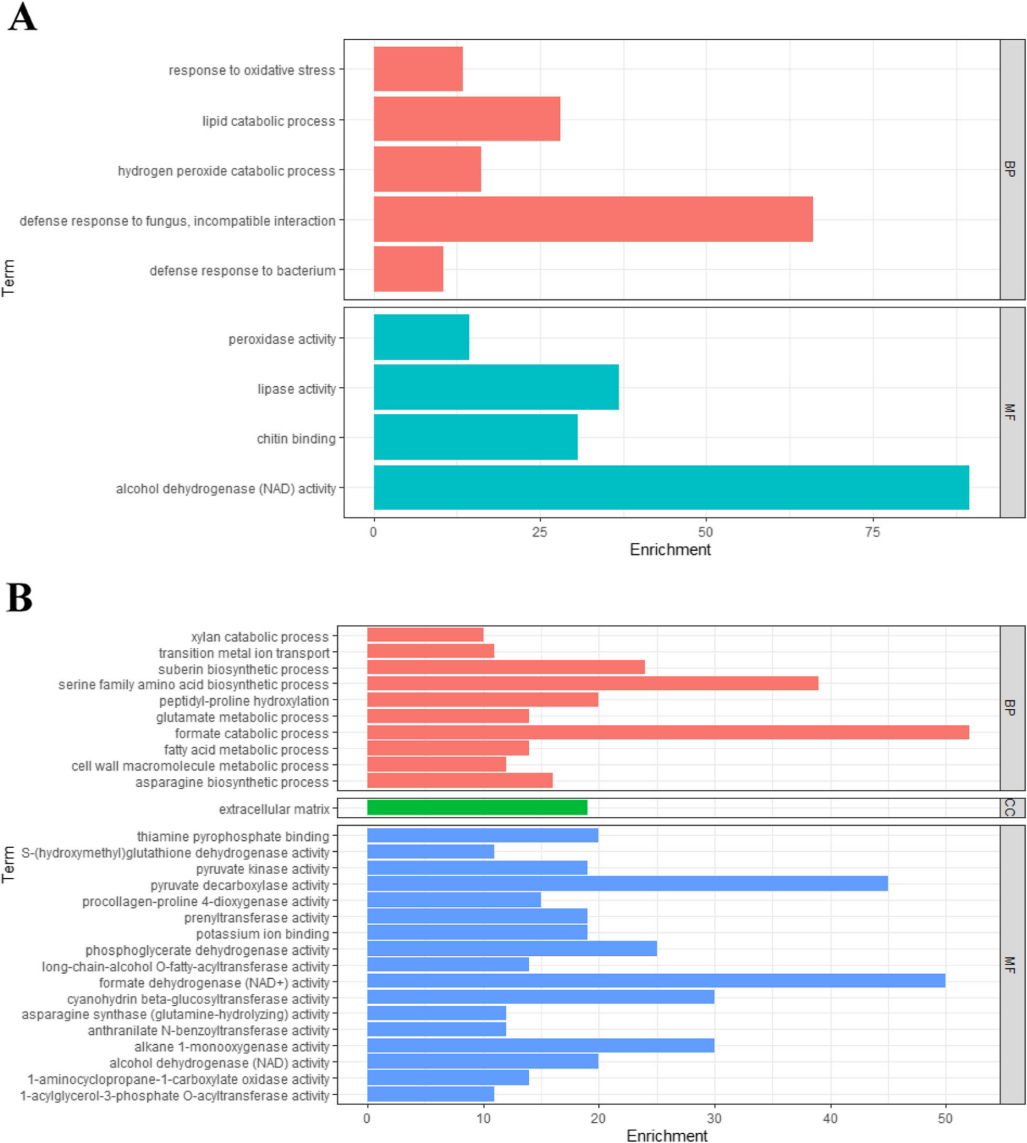
GO enrichment analysis of DEGs induced by salt stress in **A** Suzi 41 and **B** Sujiang 1419. The three GO categories-biological process (BP), cellular components (CC), and molecular function (MF)-are shown

Next, GO analysis was conducted to predict the potential functions or biological roles of these salt-induced DEGs. GO terms that were enriched among the 52 most significantly up- or down-regulated DEGs (14 in Suzi 41; 40 in Sujiang 1419) under salt stress indicated that these genes were likely related to biological processes and molecular functions. More specifically, the DEGs in Suzi 41 were enriched in biological processes such as “hydrogen peroxide catabolic process” (GO:0042744) and “response to oxidative stress” (GO:0006979), while molecular function-associated terms included “peroxidase activity” (GO:0004601). By contrast, the DEGs in Sujiang 1419 were enriched in “suberin biosynthetic process” (GO: 0010345), “transition metal ion transport” (0000041), and “peptidyl-proline hydroxylation” (GO:0019511) biological processes, while molecular function-associated terms included “alcohol dehydrogenase (NAD) activity” (GO:0004022) and “potassium ion binding” (GO:0030955). It is noteworthy that “alcohol dehydrogenase (NAD) activity” (GO:0004022) was the only term enriched in both varieties (Fig. 3, Supplementary Table S6).

**Fig. 4:**
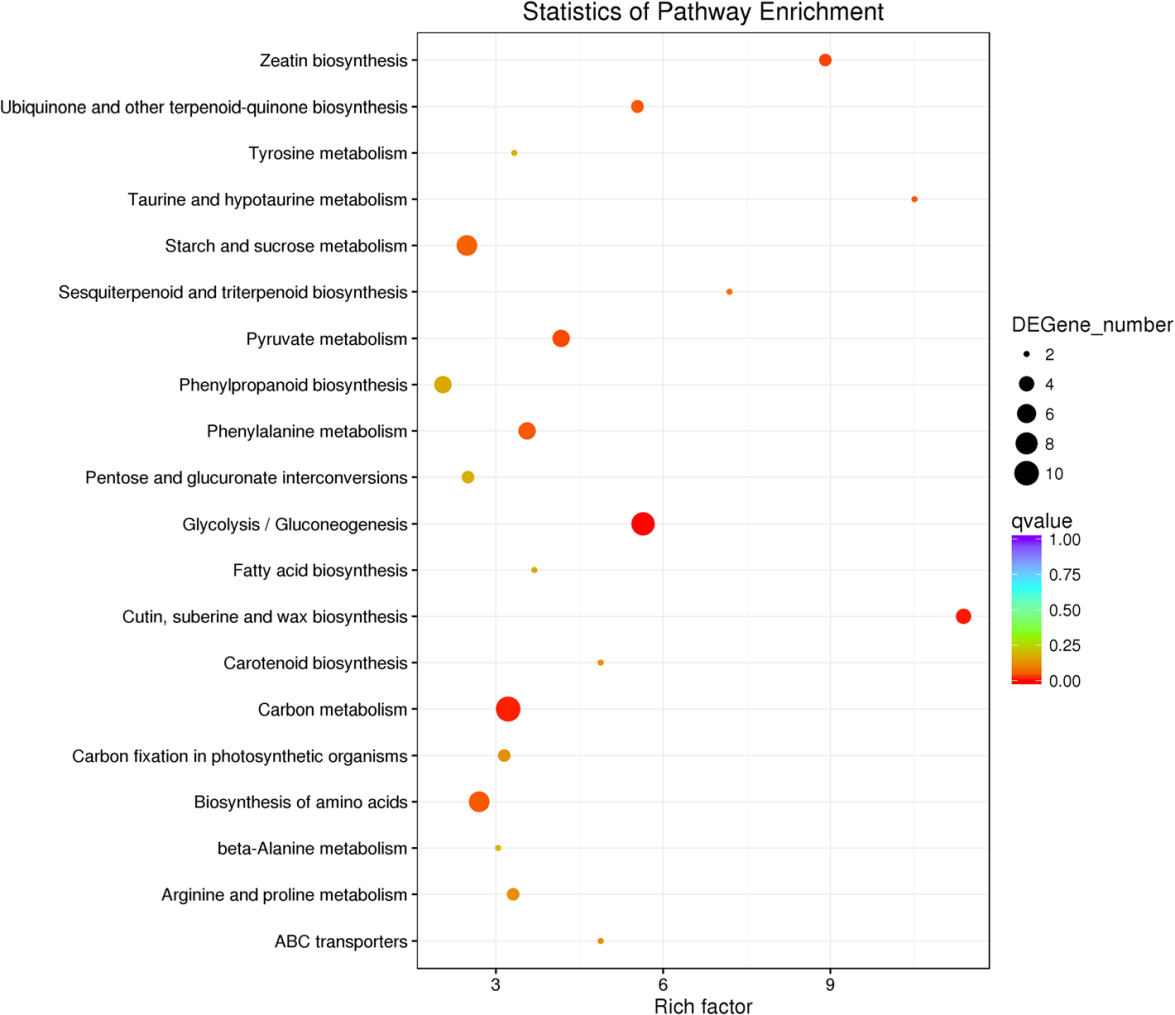
KEGG pathway enrichment analyses of DEGs under salt stress for Sujiang 1419

To identify the metabolic pathways in which the DEGs were involved and enriched, KEGG analysis was also performed [28]. The pathways enriched for the most highly up- or down-regulated significant DEGs are listed in Fig. 4. Among these pathways, “Glycolysis/Gluconeogenesis” (ko00010, *p*-value = 2.32E-05), “Cutin, suberine and wax biosynthesis” (ko00073, *p*-value = 0.0004), and “phenylpropanoid biosynthesis” (ko00940, *p*-value = 0.0126) etc., were enriched in Sujiang 1419. The only significantly enriched biological pathway found in Suzi 41 was “cysteine and methionine metabolism” (ko00270, *p*-value = 0.0032), in which an ethylene biosynthetic enzyme-encoding gene (*Vigun02g178400*), 1-aminocyclopropane-1-carboxylate synthase (ACS, EC:4.4.1.14), was significantly up-regulated (Supplementary Table S7). This finding thus suggested that ethylene signaling may contribute a major role in tolerance to salt stress for *V. unguiculata* subsp. *sesquipedalis*.

### Differential expression of transcription factors between the two varieties under salt stress

Transcription factors play crucial roles in regulating the expression of stress response genes during exposure to high salinity. A total of 224 differentially expressed TFs (47 families) were identified under salt stress in Suzi 41 and Sujiang 1419 (Supplementary Table S8). These TFs include MYB, B3, NAC, AP2/ERF, MADS, GNAT, plant basic helix–loop–helix (bHLH), C2H2, and WRKY. MYB composed the largest percentage (19 TFs, 16.38%), followed by B3 (15 TFs, 12.93%), NAC (13 TFs, 11.21%), and AP2/ERF (13 TFs, 11.21%), indicating that these TFs may be major determinants controlling the mechanisms of salt stress tolerance in yardlong bean. Several of the transcription factors, such as NAC and MYB, which are known to be induced by exposure to high salt conditions in *Arabidopsis thaliana,* halophytic *Suaeda liaotungensis,* wheat, and rice [29–32], were highly expressed under salinity stress in both Suzi 41 and Sujiang 1419.

Specifically, 17 transcription factors were found to be significantly up- or down-regulated only in salt tolerant Suzi 41 at a Log_2_ fold change of 1.0 or higher (Table 1). Three out of six of the most up-regulated TFs were hypothetical LR48 proteins including *Vigun06g162600*, *Vigun08g102200*, and *Vigun02g140100*, which were up-regulated 1.23, 1.27, and 1.47, respectively. The two most up-regulated transcription factors found only in Suzi 41, *Vigun06g141200* and *Vigun11g052100* (Log_2_ fold change = 1.47 and 1.36, respectively), are both *MADS-M-Type* TFs, the latter of which is annotated as a probable TAT2 aminotransferase. Two *AP2/ERF* genes were a WAT1-related AP2/ERF family protein (*Vigun06g228300*) and Ethylene-responsive transcription factor (*Vigun07g178200*), which had Log_2_ fold lower expression = − 1.02 and 1.04 in salt-stressed plants compared to non-stressed plants. These genes may play an important role in plant response to salt stress. In addition, three other families, ABC transporter (*Vigun02g026100*), WRKY (*Vigun01g071800*), and bHLH (*Vigun03g388100*), showed 1.14, 1.07, and 1.00, Log_2_ fold up-regulation, respectively, implying that these TFs may also participate in salt-regulated pathways. The differential expression of these genes exclusively in Suzi 41 during salt exposure suggests these genes may be apt targets for molecular breeding for increased salt tolerance in yardlong bean.

**Table 1:** Up- or down-regulated transcription factors in Suzi 41 under salt stress

Gene name	TF family	Regulated	41C_vs_41S_ log_2_FC	1419C_vs_1419S_ log_2_FC	Annotation
Vigun01g071800	WRKY	Up	1.07	No change	Probable WRKY transcription factor 75
Vigun02g026100	RWP-RK	Up	1.14	No change	ABC transporter G family member 21
Vigun02g140100	Others	Up	1.47	No change	Hypothetical protein LR48
Vigun02g174400	CSD	Up	1.08	No change	Osmotin-like protein OSM34
Vigun03g388100	bHLH	Up	1	No change	Transcription factor bHLH35
Vigun03g443100	SNF2	Up	1.1	No change	Hypothetical protein LR48
Vigun04g107600	B3- > B3	Up	1.11	No change	Cytochrome P450 81E8
Vigun06g141200	MADS- > MADS-M-type	Up	1.47	No change	Peroxidase 54
Vigun06g162600	B3- > B3	Up	1.23	No change	hypothetical protein LR48
Vigun06g228300	AP2/ERF- > AP2/ERF-ERF	Down	−1.02	No change	WAT1-related protein At1g68170
Vigun07g178200	AP2/ERF- > AP2/ERF-ERF	Up	1.04	No change	Ethylene-responsive transcription factor 1B
Vigun07g247000	GNAT	Up	1.19	No change	Cysteine-rich receptor-like protein kinase 29
Vigun08g054400	Trihelix	Up	1.12	No change	14 kDa proline-rich protein DC2.15
Vigun08g102200	mTERF	Up	1.27	No change	Hypothetical protein LR48
Vigun09g085100	bHLH	Up	1.1	No change	Uncharacterized protein LOC106761581
Vigun11g052100	MADS- > MADS-M-type	Up	1.36	No change	Probable aminotransferase TAT2
Vigun11g159900	GRAS	Up	1.01	No change	UDP-glucose iridoid glucosyltransferase

Twelve transcription factors were significantly up- or down-regulated in both Suzi 41 and Sujiang 1419 (Supplementary Table S9). Notably, *Vigun08g132400*, a hypothetical MADS-M-Type TF showed Log_2_ fold increases of 2.67 and 1.26 in salt tolerant and salt sensitive varieties, respectively. Similarly, 305-like MYB-related protein *Vigun07g057300* exhibited Log_2_ fold up-regulation of 1.50 and 2.11 in the tolerant and sensitive lines, respectively. Among differentially down-regulated TFs, MADS-M-Type *Vigun06g084400* was Log_2_ fold lower in both Suzi 41 and Sujiang 1419 at − 1.12 and − 1.93, respectively. While these genes are not indicative of a salt stress response unique to Suzi 41, their differential expression in both varieties during salt exposure suggests that they belong to potentially universal stress response pathways across yardlong bean varieties.

### qRT-PCR validation of differentially expressed genes

**Fig. 5:**
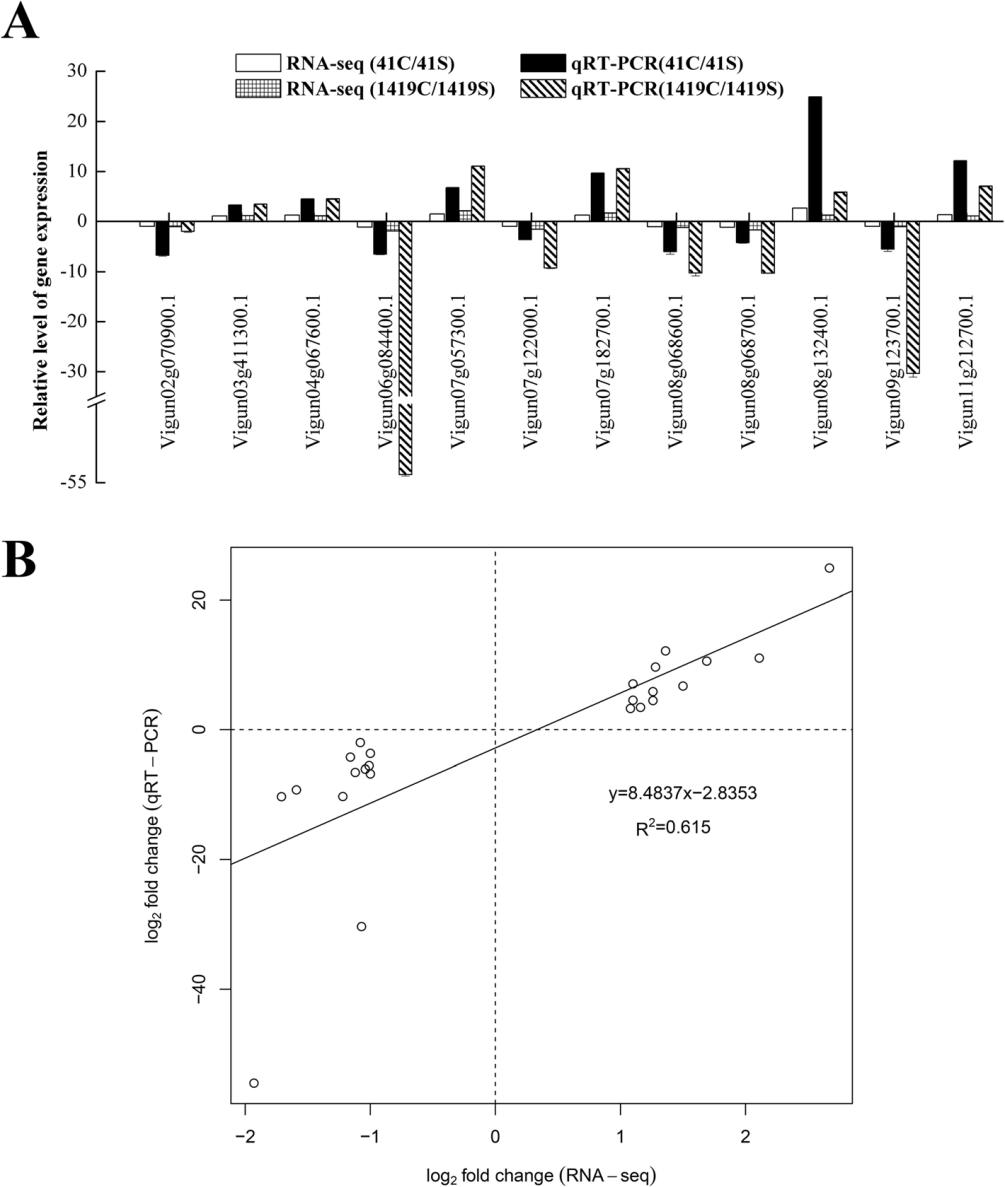
Correlation analysis of gene expression between RNA-seq and qRT-PCR. **A** Results of qRT-PCR validation of 12 DEGs in root tissue under salt treatment. All qRT-PCR reactions were performed with three biological replicates. **B** Scatterplot showing correlation between Log_2_ fold-change values obtained from 12 DEGs by RNA-seq (X-axis) and qRT-PCR (Y-axis)

To validate the results of DEG identification in the RNAseq data, qRT-PCR was used to confirm the differential up- or down-regulation for 12 randomly selected DEGs (Fig. 5A). Six of the 12 DEGs (*Vigun03g411300*, *Vigun04g067600*, *Vigun07g057300*, *Vigun07g182700*, *Vigun08g132400*, *Vigun11g212700*) were up-regulated in Suzi 41 and Sujiang 1419 during NaCl treatment, while the other six DEGs (*Vigun02g070900*, *Vigun06g084400*, *Vigun07g122000*, *Vigun08g068600*, *Vigun08g068700*, *Vigun09g123700*) were down-regulated in the two genotypes. Although the gene expression values are different using both techniques, all of these genes displayed similar expression trend. Comparison of the qRT-PCR and RNAseq data revealed a high correlation for the selected unigenes (Pearson r = 0.7835, Fig. 5B), ultimately reflecting consistency between the qRT-PCR and transcriptomic results.

### Several DEGs associated with salt tolerance are located on chromosome 11 of the yardlong bean genome

Based on our previous studies, six major QTLs associated with salt tolerance were detected in the region between VUIn584 ~ VUIn724, between VUIn282 ~ VUIn815, and between VUIn675 ~ VUIn578 on chromosomes 9, 11, and 8 [24]. In the present study, nine DEGs in the region of VUIn282 ~ VUIn815 on Chr.11 were found, eight of which were differentially expressed only in Suzi 41 under salt treatment (Table 2). Three of these DEGs encoded hypothetical LR48 proteins, *Vigun11g159900*, *Vigun11g188200* (the only one differentially expressed in both Suzi 41 and Sujiang 1419), and *Vigun11g170200*, which were up- or down-regulated 1.01, 1.10, and − 1.20, respectively. Furthermore, the two most up-regulated DEGs found only in Suzi 41, *Vigun11g186800* and *Vigun11g174500* (Log_2_ fold change = 1.69 and 1.54, respectively), are respectively annotated as a probable BOI-related E3 ubiquitin-protein ligase and an organ-specific protein. In addition, four other DEGs, aldehyde dehydrogenase family (*Vigun11g160800*), hypothetical protein (*Vigun11g170300*), myosin heavy chain kinase (*Vigun11g170300*), and putative lipid-transfer protein DIR1 (*Vigun11g172800*), showed Log_2_ fold lower expression = − 1.31, − 1.28, 1.08, and − 1.22 in salt-stressed plants compared to non-stressed plants (Table 2). The positions of these DEGs have not been previously reported, and their mapping to on Chr.11 thus provides a valuable resource for studying salt stress-induced genes in yardlong bean.

**Table 2:** Nine salt stress-induced significant DEGs on Chr.11 in *V. unguiculata*

Gene name	*P*-value	FDR	41C_vs_41S_ log_2_FC	1419C_vs_1419S_ log_2_FC	Annotation
Vigun11g159900	8.59E-06	0.002265438	1.01	No change	Hypothetical protein LR48_Vigan238s006900 [*Vigna angularis*]
Vigun11g160800	1.95E-07	2.67E-05	−1.31	No change	Aldehyde dehydrogenase family 3 member F1
Vigun11g170000	3.31E-06	0.000252395	−1.28	No change	Hypothetical protein VIGAN_06216500 [*Vigna angularis* var. angularis]
Vigun11g170200	6.58E-05	0.002717521	−1.2	No change	Hypothetical protein LR48_Vigan08g167400 [*Vigna angularis*]
Vigun11g170300	0.000180926	0.005851282	1.08	No change	Myosin heavy chain kinase B
Vigun11g172800	4.79E-07	5.34E-05	−1.22	No change	Putative lipid-transfer protein DIR1
Vigun11g174500	1.14E-07	1.71E-05	1.54	No change	Organ-specific protein S2
Vigun11g186800	4.55E-10	2.37E-07	1.69	No change	Probable BOI-related E3 ubiquitin-protein ligase 3
Vigun11g188200	1.52E-06	0.000592126	1.1	1.29	Hypothetical protein LR48_Vigan08g183200 [*Vigna angularis*]

### 26 polymorphic InDels in DEGs between Suzi 41 and Sujiang 1419 show allelic differences and high genetic diversity among yardlong bean germplasm

**Fig. 6:**
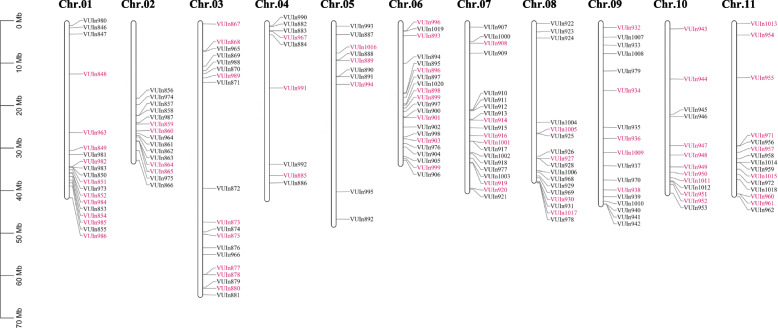
Distribution of 175 InDel markers on each chromosome in the *Vigna unguiculata* (L.) *Walp.* InDel marker names are listed to the right of the chromosomes. The ruler to the left of chromosomes represents physical distance. The red indicates deletions; black indicates insertions

**Fig. 7:**
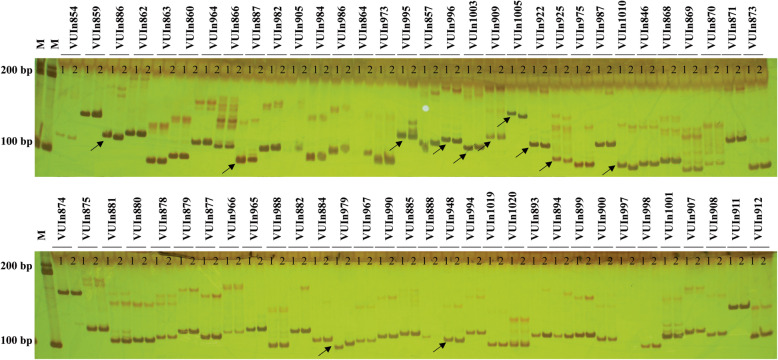
PCR screening of polymorphisms using InDel primers in the two *V. unguiculata* varieties. M: Marker, 1: Suzi 41, 2: Sujiang 1419. Black arrows show polymorphic InDels

Genome-wide identification of insertion/deletion polymorphisms was conducted via TopHat 2.0 software. In total, 175 InDels located in DEGs were identified in the RNAseq data (Fig. 6, Supplementary Table S10) in Suzi41 and Sujiang 1419, including 134 InDels in Suzi41 which were distributed across all the eleven chromosomes, varying from 17 on Chr.03 to six each on Chr.04 and Chr.05. At the same time, 147 InDels were identified in Sujiang 1419 that ranged from 20 on Chr.07 to 7 on Chr.05 (Supplementary Table S11). Among these, the largest InDel was 42 bp, although InDels smaller than 3 bp were prevalent and accounted for about 80% of the total. The proportions of InDels less than 10 bp were 94.78 and 94.56% in Suzi 41 and Sujiang 1419, respectively, and InDels smaller than 6 bp accounted for 87.31 and 89.80%, respectively (Supplementary Table S12). The genome sequences of two different varieties were used to validate the 175 InDel polymorphisms identified through RNA-seq. Among the tested markers, 26 primer pairs (14.86%) produced clear amplicons with the expected sizes in both yardlong bean varieties, while 127 primer pairs (72.57%) produced amplicons in only one genotype and therefore were not suitable for genetic analysis, 7 (4.00%) were monomorphic, and 15 (8.57%) failed to amplify altogether (Fig. 7). To further confirm whether these 26 InDel polymorphisms could be used as markers in other yardlong bean accessions, these primer pairs were validated in 84 additional germplasm accessions of yardlong bean (Supplementary Table S13). A total of 58 alleles were detected and scored (Supplementary Table S14). The genetic diversity index ranged from 0.0887 to 0.5907, and the average was 0.4366. The polymorphic information content (PIC) values ranged from 0.0848 to 0.5103, and the average was 0.3494. The markers used in this study represent a broad level of diversity in *V. unguiculata* and can effectively discriminate between many publicly available germplasm accessions*.* Overall, these InDel markers developed here reveal a high number of alleles for these DEGs and substantial genetic diversity among accessions.

## Discussion

In this study, the whole transcriptomes of salt tolerant variety Suzi 41 with that of the salt sensitive variety Sujiang 1419 were compared under high salinity conditions to determine which genes confer tolerant phenotype in *V. unguiculata* subsp. *sesquipedalis*. Using RNA-seq, transcriptional profiles were developed for salt stress responsive gene networks in salt-tolerant and -susceptible varieties. Under high salinity, stress tolerant plants apparently had fewer DEGs than sensitive plants compared with control plants. These results aligned with other transcriptomic studies of response to drought and salt stress in sorghum and *Corchorus* spp. [33, 34]. Consistent with this finding, fewer GO categories were enriched with DEGs in Suzi 41 than in Sujiang 1419 under salt stress. In Suzi 41, five and four GO terms were enriched with DEGs in the biological process and molecular function categories, respectively. Among the enriched biological processes in Suzi 41 were “hydrogen peroxide catabolic process” (GO: 0042744) and “response to oxidative stress” (GO: 0006979). Hydrogen peroxide plays an important role in stress response by coordinating intracellular and systemic signaling systems that facilitate plant acclimatization and tolerance to stress [35]. Oxidative stress results from plant inability to mitigate free radical damage with sufficient antioxidant activity under salt stress [36]. These two GO terms are only associated with the salt-tolerant yardlong bean variety and were primarily enriched with upregulated DEGs. These results suggest that salt-tolerant varieties can acclimate to salinity stress by relieving oxidative stress through the stimulation of salt resistance-related gene expression via ROS signals and regulation of redox pathways. However, there are 10, 1, and 17 GO categories enriched in biological process, cellular component and molecular function respectively in Sujiang 1419. “Alcohol dehydrogenase activity” (GO: 0004022) is the only GO category enriched (entirely with downregulated DEGs) in both Suzi 41 and Sujiang 1419. Alcohol dehydrogenase (ADH) activity is considered a necessary condition for plant survival under anaerobic conditions. The role of plant *ADH* gene in anaerobic stress response has been a long-standing focus of research [37, 38]. Our study shows that alcohol dehydrogenase may participate in the salinity stress response by yardlong bean.

By mapping these DEGs, three major QTL regions and a set of 26 InDel markers were successfully identified that could reliably genotype a panel of 84 *V. unguiculata* germplasm accessions. In addition, LR48 and ERF family transcription factors were found to potentially play a major role in tolerance to salt stress given their prevalence among the highest up-regulated DEGs in Suzi 41. Finally, genetic diversity was assessed through PIC analysis and validated InDel markers for salt response using a wide panel of yardlong bean germplasm accessions.

Although few significant DEGs were correlated with high salt treatment in the Suzi 41 variety, comparisons between high salinity treatment and control plants revealed strong induction of *Vigun07g178200* (ethylene-responsive transcription factor 1-*ERF1*), suggesting its participation in a tolerant response to salinity. *ERF1* up-regulation has been widely described as a step in the ethylene-mediated salt stress response signal cascade [39–41]. Importantly, *AP2/ERFs* are widely known to regulate hormone signaling and abiotic stress response pathways, as reviewed by Xie et al. 2019 [42]. For example, transgenic expression of *GmERF3* from soybean in *Nicotiana tabacum* enhanced its tolerance to salinity, as well as other abiotic stresses [43]. In addition, *Vigun02g026100* (ABC transporter G family member 21) was differentially up-regulated in Suzi 41 (Table 1). In plants, ABC transporters are typically associated with heavy metal detoxification [44] or with auxin transport [45, 46]. Another recent investigation of salt stress response in *V. unguiculata* reported similar numbers of DEGs for tolerant and sensitive varieties (i.e.*,* 13 DEGs from six different TF families) and identified 17 SNP markers associated with six salt-induced DEGs [23]. Similarly, our study also found six major QTLs across chromosomes 8, 9, and 11, one of which contained three differentially expressed LR48 family TFs.

Interestingly, the association of these three salt-inducible LR48 transcription factors (*Vigun11g159900*, *Vigun11g170200*, and *Vigun11g188200*) suggested that these genes could contribute a potentially important role in tolerance to salt stress in yardlong bean. Although surprisingly little has been reported on the structure, domains, or mechanistic function of the LR48 gene, it is commonly used in marker-assisted breeding to confer hypersensitive response-mediated resistance to Leaf Rust (*Puccinia triticina*) in wheat [47–49]. In addition to potential disease resistance, which requires further study, the preponderance of *LR48-like* genes in our dataset strongly suggests that they could function in abiotic stress response in *V. unguiculata*. In addition to the LR48 and ERF transcription factors, a bHLH35 transcription factor (*Vigun03g388100*), a WRKY75 transcription factor *(Vigun01g071800*), and an AP2-ERF ethylene-responsive transcription factor (*Vigun07g178200*) were identified among the highly significant, Suzi 41-specific salt-inducible DEGs.

In agreement with our findings that AP2-ERF, WRKY and bHLH transcription factors for their critical role in osmotic stress signaling mediated by salt [8, 50–52]. Previous studies in *Arabidopsis* have revealed the detailed regulatory role WRKY8 in modulating tolerance to salt. Specifically, WRKY8 was found to bind downstream stress response genes under salt exposure, and its knockout resulted in an increased Na^+^/K^+^ ratio, hypersensitivity to salt, and other developmental abnormalities [53]. In addition, bHLH transcription factors, such as AtbHLH112 in *Arabidopsis*, mediate tolerance to high salinity. In this example, AtbHLH was shown to localize to the nucleus and bind GCG- and E-box motifs in target gene promoters during salt and drought treatment. Its functionality was correlated with enhanced salt tolerance, higher proline levels, and elevated expression of POD and SOD genes to mitigate ROS damage [54]. Notably, *bHLH* genes have been shown to affect plant physiological response to stress, such as *SlbHLH22,* which was found to be elevated under high salinity- or D-mannitol-induced stress in tomato [55]. The prevalence of *LR48*, *bHLH*, *WRKY*, and *AP2-ERF* TFs among the stress-induced DEGs suggests that these genes may serve as promising targets for improvement of salt tolerance in *Vigna unguiculata.*

In addition to marker development and transcriptomic profiling, genetic diversity analysis was conducted as a necessary step in accessing the full potential of these genomic and transcriptomic resources. Molecular markers are effective tools to evaluate the genetic diversity, and disclose the evolution history of cowpea resources. For example, Asare et al. (2010) analyzed the genetic diversity of 141 cowpea in Ghana using 20 pairs of SSR primers; Xiong et al. (2016) investigated the genetic polymorphism of 784 cowpea genotypes worldwide using SNP markers, and predicted the migration and domestication history of cowpea [56, 57]. In our study, the InDel markers based on high-throughput transcriptome sequencing are different from the molecular makers based on DNA polymorphism, since they locate in coding sequences that directly related to the phenotype characters. A total of 175 InDel markers were developed in our research, 26 of which were used to evaluate the genetic diversity of 84 yardlong bean accessions. A high level of diversity in *V. unguiculata* was found by using the InDel markers developed here. Collectively, these results indicated that the InDels not only can serve as effective markers, comparable to SSRs, for estimating genetic diversity in yardlong bean, but also provide a basis for future research on gene function.

## Conclusions

This transcriptomic analysis of salt-inducible genes in yardlong bean revealed a suite of candidate DEGs largely comprised of *ERF*, *LR48*, *WRKY*, and *bHLH* transcription factors. A robust set of 26 salt stress-related InDel markers were developed that can be used for improvement of salt tolerance in yardlong bean germplasm. Finally, genetic diversity analysis in a wide panel of accessions was performed which demonstrated the effectiveness and reliability of these InDel markers. This work therefore provides a genetic resource for improving yields in low quality soils, and offers foundational insights into the basic mechanisms underlying abiotic stress response in *V. unguiculata*.

## Methods

### Plant materials

The seeds for this study contained salt-tolerant Suzi 41 and salt-sensitive Sujiang 1419 [24], and 84 other yardlong bean accessions, among which 77 were collected from the National Infrastructure for Vegetable Crop Germplasm Resources (NIVCGR), and 7 from Jiangsu Academy of Agricultural Sciences (JAAS) (Supplementary Table S13).

### Plant growth and salt stress treatments

Two yardlong bean genotypes: salt-tolerant Suzi 41 and salt-sensitive Sujiang 1419 [24] were employed to examine differences in the expression of genes involved in a tolerant response to high salinity. Ten seeds of Suzi 41 and Sujiang 1419 were sown in cups (9.5 × 16 cm) filled with vermiculite, with three replicates per genotype. Following germination, four plants of each variety were placed in a plastic tank (50 × 40 × 20 cm) filled with aerated half-strength Hoagland nutrient solution [58] and allowed to grow until the true leaves fully expanded. Four seedlings were then placed in half-strength Hoagland nutrient solution containing 50 mM NaCl for 1 day. The concentration was raised by adding 50 mM NaCl each day until a final concentration of 150 mM was reached. At the same time, untreated seedlings were transferred to a tank filled with half-strength Hoagland nutrient solution without added salt to serve as the control. The roots were harvested separately after 3 days of treatment. Each sample was derived from at least four individual plants with three biological replicates per genotype for each treatment. The plant roots were frozen in liquid nitrogen and kept at − 80 °C.

### RNA-seq

Total RNA was isolated using the RNAprep Pure Plant Kit (Tiangen Biotech Co., Ltd., Beijing, China) according to the manufacturer’s protocols. RNA samples were then visualized on a 1% agarose gel and quantified with a NanoDrop ND-1000 spectrophotometer (Thermo Fisher Scientific, Inc. Waltham, MA, USA). Twelve root samples (Suzi 41 salt-stressed, 41S; Sujiang 1419 salt-stressed, 1419S; Suzi 41 control, 41C; Sujiang 1419 control, 1419C) were used for transcriptome sequencing by the Biomarker Biotechnology Corporation (Beijing, China). For each sample, three independent biological replicates were performed.

RNA-seq libraries were prepared using the paired-end strategy. In detail, (1) Poly (A) mRNA was enriched using the NEBNext® Poly (A) mRNA Magnetic Isolation Module (New England Biolabs, Ipswich, MA, USA), and then it was fragmented into short pieces chemically. (2) The first- and second-strand cDNA were synthetized using the short mRNA as template and then subjected to end-repair and phosphorylation using T_4_-DNA polymerase and Klenow DNA polymerase. The repaired cDNA fragments were inserted ‘A’ bases as overhangs at the 3′ ends and connected with sequencing adapters. (3) The suitable fragments were selected for the PCR amplification as templates after agarose gel electrophoresis. Finally, the twelve libraries were sequenced using Illumina HiSeq™ 2000 sequencing system.

### Annotation

After RNA-seq, the raw data were purified by trimming adapters and removing low-quality sequencing to get clean reads. At the same time, Q20, Q30 and GC content of the clean data were calculated. Reference genome and gene annotation files were downloaded from the website (https://phytozome.jgi.doe.gov). All clean reads were matched to the *Vigna unguiculata* reference genome by TopHat v2.0 [59].

### Analysis of differentially expressed genes

The expression levels of genes were calculated using the FPKMs (fragments per kilo-base of exon per million fragments) method, which were computed by summing the FPKMs of transcripts in each gene group [60]. Here, only fold change with an absolute value of *P* ≤ 0.01 and |log_2_(ratio)| ≥ 1 were used as the threshold for significant differential expression and for subsequent analysis.

### Differential gene functional annotation

The KEGG pathways were analyzed for differentially expressed genes and the corresponding ko numbers were predicted using KOBAS software [61]. A statistical analysis of the GO (Gene Ontology) term for genes in the biological process, cellular component, and molecular function classifications was implemented by the GOseq R package (1.10.1) [62], in which gene length bias was corrected. GO terms with *p* < 0.05 were considered significantly enriched by differentially expressed genes.

### Validation by real-time PCR (qRT-PCR)

In order to validate the reliability of RNA-seq experiments, a total of 12 DEGs were randomly selected for qRT-PCR analysis of relative expression. Sequences of the specific primers used for qRT-PCR are given in Supplementary Table S15. A total of 0.5 μg of DNaseI-treated total RNA was converted into single-stranded cDNA using a Prime-Script 1st Strand cDNA Synthesis Kit (TaKaRa, Dalian, China). The cDNA templates were then diluted 20-fold before use. The quantitative reaction was performed on a CFX96 Real-Time PCR Detection System (Bio-Rad, Singapore) using SYBR Premix Ex Taq™ (TaKaRa, Dalian, China). PCR amplification was performed under the following conditions: 30 s at 95 °C, followed by 40 cycles of 95 °C for 5 s, 60 °C for 20 s and then 72 °C for 20 s. The relative expression levels of the selected DEGs normalized to an internal reference gene were calculated using the 2^-ΔΔCt^ method [63]. The UBC9 housekeeping gene (*Vigun05g084500*) was used as the internal reference, and all analyses were performed with three technical and three biological replicates.

### Identification of short InDels and primer design

The transcriptomic sequencing results of salt-tolerant Suzi 41 and salt-sensitive Sujiang 1419 were mapped to the cowpea reference genome (https://phytozome.jgi.doe.gov) using TopHat 2.0 [64]. With reads from each genotype and treatment mapped to the reference genome, GATK v3.5 [65] was used to call SNPs and InDels for each sample. After filtering out unreliable sites, the final set of SNPs and InDels in VCF format were obtained. To develop single-product, amplifiable InDel markers, primers were designed to amplify each InDel with 150 bp 5′ and 3′ flanking sequence using Primer 3.0 [66] with a total amplicon lengths between 100 and 250 bp. The primers were constrained by a required melting temperature between 58 and 65 °C, primer optimal length of 20 nt (18–25 nt), minimum GC of 40%, maximum GC of 65%, and optimized GC at 50%. To avoid selection of microsatellites around InDels, only those InDels in which none of the flanks contained microsatellites identified by MISA [67] with default parameters, were used for further primer design.

### DNA extraction and PCR

Genomic DNA of each yardlong bean accession was extracted from young leaves using the Hexadecyltrimethylammonium bromide (CTAB) method [68]. A Nano-Drop 2000 1spectrophotometer (Nano Drop Technologies, USA) was used to evaluate the quality and concentration of all DNA. DNA samples were diluted to 25 ng/μL. PCR was performed in a total volume of 10 μl containing 50 ng of genomic DNA, 0.4 U of Taq DNA polymerase (Dingguo Biological Technology Development Co., Ltd., Beijing, China), 10× Taq Buffer II, 25 mM MgCl_2_, 2 mM of dNTPs, and 1 mM each forward and reverse primer. PCR conditions were as follows: 94 °C for 4 min, 30 cycles of 30 s at 94 °C, 52 ~ 58 °C for 30 s, 72 °C for 30 s, and 1 cycle at 72 °C for 2 min. The PCR products were separated on an 8.0% non-denaturing polyacrylamide gel electrophoresis (PAGE) gel and then visualized by silver staining. DL2000 Marker DNA ladder (TsingKe, Biological Technology Co., Ltd., Nanjing, China) was used as the standard size marker.

### Chromosomal location

The chromosomal location of InDel markers was acquired from the cowpea genome database (https://phytozome.jgi.doe.gov) as a reference genome, and the InDel markers were mapped onto chromosomes using MapDraw [69].

### Data analysis

PCR products were scored manually, and a 0/1 binary matrix was set according to the presence or absence of corresponding amplified bands. Genotypic genetic diversity analysis used PowerMarker V3.25 (http://www.powermarker.net) [70] to obtain allele frequency, allele number, gene diversity index, and genotype polymorphism information content (PIC).

## Supplementary Information

**Additional file 1: Supplementary Table S1.** Number of reads obtained by RNA sequencing and their matches in the *Vigna unguiculata* genome.

**Additional file 2: Supplementary Table S2.** Summary of novel transcript isoforms found in *V. unguiculata* genomic data.

**Additional file 3: Supplementary Table S3.** Differentially expressed genes (DEGs) in Suzi 41 and Sujiang 1419 under salt stress.

**Additional file 4: Supplementary Table S4.** Significant DEGs exclusive to Suzi 41 and Sujiang 1419 during salt stress.

**Additional file 5: Supplementary Table S5.** Differentially expressed genes that are up- or down-regulated in both Suzi 41 and Sujiang 1419 during salt stress.

**Additional file 6: Supplementary Table S6.** GO analysis of DEGs induced by salt stress in Suzi 41 and Sujiang 1419.

**Additional file 7: Supplementary Table S7.** KEGG pathways enriched with DEGs under high salinity in Suzi 41 and Sujiang 1419.

**Additional file 8: Supplementary Table S8.** TF genes identified as DEGs in Suzi 41 and Sujiang 1419 under salt stress.

**Additional file 9: Supplementary Table S9.** TFs that are up- or down-regulated in both Suzi 41 and Sujiang 1419.

**Additional file 10: Supplementary Table S10.** InDels identified on individual chromosomes of yardlong bean.

**Additional file 11: Supplementary Table S11.** The number and distribution ratios of InDels identified in yardlong bean.

**Additional file 12: Supplementary Table S12.** Characteristics of 175 InDels derived from RNA-seq data of salt-stressed yardlong bean.

**Additional file 13: Supplementary Table S13.** List of 84 yardlong bean germplasm accessions used in this study.

**Additional file 14: Supplementary Table S14.** Genetic diversity of yardlong bean based on 26 InDel markers.

**Additional file 15: Supplementary Table S15.** Primer used for qRT-PCR analysis.

## Data Availability

We have deposited our data in Sequence Read Archive (SRA) (http://www.ncbi.nlm.nih.gov/sra/), the accession number for our submissions are: PRJNA388018.

## Abbreviations

RNA-seq: RNA sequencing
TFs: Transcription factors
AKT1: *Arabidopsis* K^+^ transporter1
NHXs: Na^+^/H^+^ exchangers
HKT: High sodium affinity transporter
PMP: Plasma membrane proteins
AP2/ERF: Apetala2/ethylene responsive factor
DREB: Dehydration responsive element binding protein
bZIP: Basic leucine zipper domain
MAPK: MAP kinase
GWAS: Genome-wide association study
41S: Suzi 41 salt-stressed
1419S: Sujiang 1419 salt-stressed
41C: Suzi 41 control
1419C: Sujiang 1419 control
PIC: Polymorphic information content
bHLH: Basic helix-loop-helix
FPKMs: Fragments per kilo-base of exon per million fragments
GO: Gene Ontology
qRT-PCR: Quantitative real-time PCR
NIVCGR: National Infrastructure for Vegetable Crop Germplasm Resources
JAAS: Jiangsu Academy of Agricultural Sciences
CTAB: Hexadecyltrimethylammonium bromide
PAGE: Polyacrylamide gel electrophoresis
PIC: Polymorphism information content
